# The Role of Bone-Derived Osteocalcin in Testicular Steroidogenesis: Contributing Factor to Male Fertility

**DOI:** 10.3390/diseases12120335

**Published:** 2024-12-20

**Authors:** Izatus Shima Taib, Putri Ayu Jayusman

**Affiliations:** 1 Centre of Diagnostics, Therapeutics and Investigative Studies, Faculty of Health Sciences, Universiti Kebangsaan Malaysia, Jalan Raja Muda Abdul Aziz, Kuala Lumpur 50300, Malaysia; izatusshima@ukm.edu.my; 2 Department of Craniofacial Diagnostics and Biosciences, Faculty of Dentistry, Universiti Kebangsaan Malaysia, Jalan Raja Muda Abdul Aziz, Kuala Lumpur 50300, Malaysia

**Keywords:** uncarboxylated osteocalcin, osteoblast, testosterone, testicular steroidogenesis

## Abstract

Osteocalcin (OCN), a protein predominantly produced by osteoblasts in bone, has emerged as a significant factor in bone metabolism and reproductive function. This article reviews the latest research on the role of OCN beyond its traditional functions in bone mineralisation, particularly its influence on testicular steroidogenesis and male fertility. The structure and modifications of OCN are elaborated upon, highlighting its uncarboxylated form (ucOCN), which is becoming increasingly recognised for its bioactive properties. The impact of OCN on bone quantity, quality and strength is summarised, emphasising its role as a regulator of bone metabolism. Furthermore, the influence of ucOCN on testicular steroidogenesis and the involvement of GPRC6A, a G protein-coupled receptor, in mediating these effects are also explored. Evidence suggests that ucOCN regulates testosterone synthesis and spermatogenesis, which indirectly have the potential to influence bone metabolism integrity. In conclusion, OCN, particularly in its uncarboxylated form, plays a crucial role in bone metabolism and male fertility by regulating testicular steroidogenesis, with GPRC6A mediating these effects, thereby linking bone health and reproductive functions.

## 1. Introduction

Osteocalcin (OCN), a non-collagenous protein, has traditionally been recognised for its role in bone metabolism and mineralisation. OCN exists in two distinct forms: carboxylated OCN (cOCN) and uncarboxylated OCN (ucOCN). OCN plays vital functions in bone health and skeletal development, serving as a key indicator of bone turnover in clinical practice [1]. The carboxylation process of OCN, which is dependent on vitamin K, enhances its binding affinity to the bone matrix [2]. Meanwhile, cOCN serves a crucial role in promoting bone mineralisation, maintaining structural integrity and regulating the balance of bone remodelling processes [3,4]. It enhances the deposition of minerals essential for achieving optimal bone strength, in addition to facilitating the recruitment and differentiation of osteoclasts and osteoblasts during the remodelling cycle to maintain an adequate balance between bone formation and bone resorption. Previous studies reported that the elevation of ucOCN is associated with lower bone mineral density (BMD) and increased risks of osteoporosis and osteopenia [5]. These findings suggest the potential role of ucOCN in regulating bone homeostasis. Aside from this, high ucOCN levels can indicate poor vitamin K status in the body, which is linked to poor bone status [6].

Recent investigations have uncovered the multifaceted roles of OCN, particularly its uncarboxylated form, in various biological processes. This protein has garnered attention beyond its traditional role in bone metabolism. ucOCN is present in the bloodstream and is believed to play various functions in many physiological processes, including glucose metabolism and energy regulation [7,8,9]. Notably, ucOCN has emerged as a key player in the regulation of testicular steroidogenesis, a process that is crucial for the synthesis of sex steroids, including testosterone [10]. In the context of male reproductive health, testicular steroidogenesis is a critical process regulated by the hypothalamic–pituitary–gonadal (HPG) axis and signalling molecules, with Leydig cells playing a pivotal role in synthesising androgens, primarily testosterone [11,12]. Emerging evidence suggests that ucOCN may modulate steroidogenesis by influencing Leydig cell function and testosterone production. This interaction highlights a potential link between metabolic status and reproductive function, as ucOCN levels are influenced by factors like insulin sensitivity and body visceral fat distribution [13], necessary for optimal testicular function [14].

Understanding the mechanisms by which ucOCN impacts testicular steroidogenesis is vital, especially considering the rising prevalence of metabolic disorders and their implications for male fertility. Recent studies have illuminated the interplay between bone and testicular function, revealing that OCN is not only crucial for bone metabolism [15] but also has a potential function in regulating testosterone synthesis [9] and spermatogenesis [16]. This connection highlights the significant role of OCN in bone health and male fertility. This review article aims to summarise the role of OCN in bone quality and strength, as well as its regulatory effects on testosterone production and male fertility. By elucidating the interplay between OCN, bone and the testis, insights into potential therapeutic targets for addressing conditions related to skeletal and reproductive health can be gained through this review.

## 2. Osteocalcin: Structure and Modifications

OCN is the most abundant non-collagenous and osteoblast-specific protein found within the bone matrix. It was first isolated and characterised by Hauschka and Price in the 1970s [17,18]. This small protein makes up approximately 1% of the total body protein, with a molecular weight of about 5.6 kDa. The structure and modification of OCN play a pivotal role in its functionality within the body. OCN features a distinctive structure characterised by three gamma-carboxyglutamic acid (Gla) residues [17,18]. The conversion of specific glutamic acid residues to Gla is essential for the function of OCN. Following protein translation at the endoplasmic reticulum, OCN is carboxylated at specific glutamic acid (Glu) residues (17, 21, and 24) by the enzyme γ-glutamyl carboxylase. This modification is vitamin K-dependent and is crucial for effective calcium binding [19,20]. Residues of pro-OCN are carboxylated to Gla residues after the removal of the 23-residue signal peptide during translation, facilitated by γ-glutamyl carboxylase (GGCX) and reduced vitamin K. Mature OCN, the 49-residue amino acid peptide, is then formed through the cleavage of a 26-residue propeptide by the proprotein convertase furin and is subsequently released into the extracellular space.

**Figure 1:**
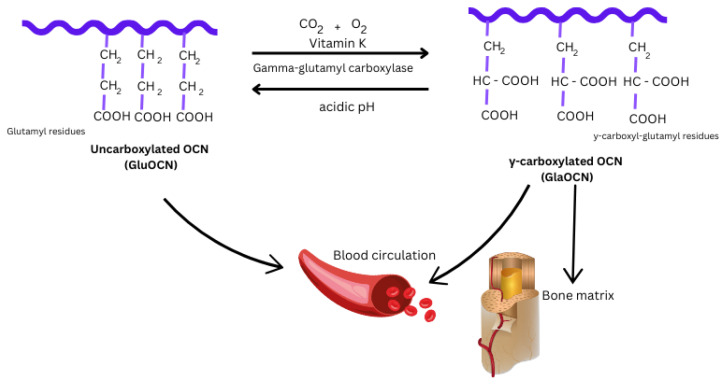
Post-translational modification of specific glutamic acid residues in OCN. The γ-carboxylation of glutamyl residues by γ-glutamyl carboxylase is catalysed by vitamin K as a cofactor.

Following post-translational modifications, most of the OCN becomes fully carboxylated and is either incorporated into the bone extracellular matrix with Ca^2+^ or released into the bloodstream (Figure 1). The carboxylation of OCN results in a conformational change that enhances its binding affinity for Ca^2+^, allowing it to specifically and strongly bind to hydroxyapatite (HA), the principal mineral component of bone. This binding supports the growth and maturation of mineral crystals in bone, highlighting its well-established role in promoting bone mineralisation and regulating metabolic processes. Meanwhile, decarboxylation of OCN occurs in the acidic environment generated by osteoclasts during bone resorption. The uncarboxylated form of OCN, known as ucOCN, has a reduced affinity for the bone matrix, allowing it to be released into the bloodstream to exert multiple effects on different tissues and organs [21]. In the last decade, pharmacological evidence has pointed out the hormonal role of this protein [22]. Most research indicates that the endocrine effects of OCN are primarily attributed to its uncarboxylated form.

## 3. The Relationship Between Testosterone and Bone Health: The Role of OCN

OCN has been traditionally recognised as a key marker of bone turnover, providing valuable insights into skeletal health and the dynamics of bone remodelling [23]. In clinical practice, OCN serves as a valuable biomarker for diagnosing bone metabolic diseases, providing information about bone tumour activity and metastasis, as well as monitoring cancer treatment efficacy [24,25]. The role of OCN in bone formation and resorption has been documented in numerous studies, along with its impact on bone quality and strength. The study conducted by Ducy et al. in 1996 was the first to provide evidence that OCN is a key determinant of bone formation [26]. Histomorphometry studies conducted in OCN knockout mice (Ocn−/−) indicated that the absence of OCN resulted in increased bone formation, bone mass and bone strength without impairing bone resorption. Contrary to expectations, the findings of the study demonstrated that bone formation was not inhibited in OCN knockout mice. A later study investigating the role of OCN in the extracellular matrix through Fourier-transform infrared imaging (FTIR) in OCN-deficient mice revealed an increase in hydroxyapatite crystal size [27], which further highlights the influence of OCN on the mineralisation process within the bone matrix.

In another study, Lambert et al. [28] showed that the complete loss of OCN in a model of OC-null rats created by the CRISPR/Cas9 system resulted in increased trabecular bone thickness, density and volume, supporting the presumption that OCN serves as a negative regulator of the skeleton. More recently, Berezovska et al. [29] explored the role of OCN in influencing the mineral and mechanical properties of bone in female mice with a pure B6 genetic background. Consistent with earlier research, it was found that six-month-old female Ocn−/− mice exhibited increased bone formation markers without any change in resorption compared to their wild-type counterparts (Ocn+/+). The study also reported that the Ocn−/− bones displayed a more immature mineral phase, higher carbonate content and lower crystallinity than the Ocn+/+ bones, as well as a reduced BMD and a lower mineral-to-matrix ratio. This observation indicated that osteoblasts in the Ocn−/− mice produced more bone matrix than their wild-type counterparts. Such findings suggest a notable imbalance in bone turnover, characterised by increased bone formation without a corresponding increase in resorption in the Ocn−/− bones. This resulted in weaker bone material qualities, leading to decreased stiffness and strength in the Ocn−/− bones. Another study suggested that removing OCN might induce structural adaptations to achieve bone strength similar to that of wild-type mice, despite a lower material quality [30].

Studies on the role of OCN in bone quality and strength using a mouse model of a double-knockout allele for Bglap and Bglap2 created through CRISPR/Cas9-mediated gene editing are inconsistent with prior results [31]. Mice with Bglap and Bglap2 double-knockout alleles did not have increased bone mass or strength. Thus, a model of OCN−/− mice with deletions of Bglap and Bglap2 was generated to resolve the controversies related to the bone phenotypes and extraskeletal functions of OCN [32]. The study showed that the collagen fibres were normally aligned in OCN-deficient mice; however, the crystallographic orientation of the biological apatite c-axis, which is normally aligned parallel to collagen fibrils, was significantly disrupted, leading to disorganised mineralisation and decreased bone strength. The findings of the study demonstrated that OCN is necessary for optimal bone quality and strength by adjusting the alignment of BAp crystallites parallel to collagen fibrils [32]. The absence of OCN led to decreased crystal thickness and impaired crystal orientation in the bones of Ocn−/− mice, suggesting the function of OCN as a major determinant in regulating the physical properties of bone mineral [33].

To summarise, the examination of skeletal traits in reported OCN knockout animal models revealed a mix of consistent and inconsistent findings. Some studies consistently demonstrate alterations in bone density, mineralisation and overall skeletal integrity, supporting the role of OCN as a key regulator of bone health. However, other research presents conflicting results, with variations in bone parameters depending on factors such as the genetic background, age and sex of the experimental animal. These discrepancies highlight the complexity of OCN’s functions, thus demanding further investigation to fully understand its effects on skeletal traits in different contexts.

## 4. Testosterone Effects on Bone Health

Testosterone is well known for its essential role in the development and maintenance of male reproductive function, yet it is equally important for promoting bone health. Testosterone exerts anabolic effects on the skeleton through two key mechanisms: by binding to androgen receptors (ARs) and by being converted to 17-beta oestradiol (E2) via the enzyme aromatase, a member of the cytochrome P450 family, which then interacts with oestrogen receptors (ERs) [34]. An investigation of the interplay between bone and gonads has been demonstrated by various study models to provide knowledge about this complex relationship. Animal models, particularly orchiectomised (ORX) rodents, have been extensively used to investigate the impact of testosterone deficiency on bone metabolism and overall skeletal health. The ORX model is utilised to simulate male osteoporosis resulting from hypogonadism, which is a major risk factor for this condition [35]. This approach is relevant since osteoporosis is recognised as a growing concern among the elderly male population. Studies are primarily performed by evaluating changes in bone density, microarchitecture and biochemical markers after removing the testes [36,37]. In addition to the ORX model, AR transgenic and AR knockout (ARKO) mouse models provide valuable information on the interaction between bone and gonadal hormones, particularly concerning bone homeostasis [38].

For instance, it was observed in a study involving ORX mice that serum levels of OCN and its uncarboxylated form were significantly decreased 16 weeks after an orchiectomy [24]. These findings indicate a relationship between testosterone and OCN, reflecting the complex interplay between the two. In another study, an increase in serum OCN was observed in young castrated rats at week 12 following an orchiectomy, indicating that this increase was associated with high bone turnover [36]. In the same study, femoral and lumbar BMDs were decreased in the ORX groups. The decrease in BMD was attributed to increased bone turnover, where there was an imbalance between bone resorption and bone formation. ORX led to significant bone loss in the femur and vertebrae, accompanied by deterioration of the microarchitecture [39]. Meanwhile, trabecular bone damage and femoral strength were diminished in the ORX group; nevertheless, testosterone replacement therapy improved femoral strength and positively influenced biomechanical parameters [40]. These findings highlighted the detrimental effects of testosterone deficiency on bone metabolism and the importance of testosterone in maintaining skeletal integrity.

Studies have shown that ARKO models exhibit significant alterations in bone density and structure, demonstrating the critical role of androgens in maintaining bone mass and skeletal integrity. Over the last few decades, ARKO models have been employed in skeletal studies [38,41], and AR inactivation in adult ARKO male mice showed a high bone-turnover bone loss. The inactivation of pre-pubertal and post-pubertal AR led to a significant decrease in total body BMD, as well as reduced tibial diaphyseal cortical bone thickness and proximal metaphyseal trabecular bone volume fraction [42]. The inactivation of AR affected trabecular bone mass, bone stiffness and bone strength in ARKO male mice [43]. These findings proved that AR is essential for maintaining trabecular and cortical bone and that its expression is vital for sustaining bone mass.

The impact of testosterone on bone health in humans is mainly assessed by measuring BMD and fracture risk. A recent study involving 5540 participants aged 20 to 59 revealed a positive association between lumbar BMD and serum testosterone levels [44]. The study also suggested that increasing testosterone levels could improve skeletal health in young and middle-aged men with low testosterone levels. Additionally, testosterone treatment consistently improved BMD at various skeletal sites, further emphasising its role in preventing osteoporosis and fractures in older populations. Drawing on discoveries from recent studies and findings from previous years, albeit with some conflicting results [45,46], testosterone supplementation therapy is believed to improve bone mass in patients with testosterone deficiency (TD). Sex hormones are crucial not only for building bone mass but also for maintaining bone mass. The influence of sex steroids on bone health is also evident in the context of androgen deprivation therapy (ADT). A study revealed that patients undergoing ADT faced an elevated risk of developing osteoporosis and experiencing osteoporotic fractures, further highlighting the crucial role of testosterone in maintaining BMD and overall musculoskeletal health in older men [47].

## 5. Uncarboxylated Osteocalcin and Testosterone Synthesis

OCN has emerged as a key regulator of male fertility and has been proposed as a potential biomarker for assessing male infertility [48]. A population-based study involving middle-aged men with moderate-to-severe erectile dysfunction (ED) found that OCN may provide protective effects against ED. This suggests that OCN could play a role in alleviating the severity of ED, potentially improving erectile function in affected individuals [49]. Additionally, an in vivo study demonstrated that Ocn−/− mice exhibited reduced testis weight, lower testosterone levels, and a decreased sperm count [50]. These effects are largely attributed to impaired testosterone synthesis in the Leydig cells of the testes, highlighting its potential role in maintaining reproductive function.

ucOCN, a form of the bone-derived protein OCN, has emerged as a significant factor in the regulation of various metabolic processes beyond its traditional role in bone metabolism. A previous study found that ucOCN interacts within the testes of mice by binding to G protein-coupled (GPRC6A) receptor [51]. GPRC6A is highly expressed in Leydig cells, and the activation of this receptor enhances the synthesis of testosterone, thus influencing male fertility [52,53]. In addition, ucOCN regulates the expression of cytochrome p450 (CYP450) and hydroxysteroid dehydrogenase (HSD) enzymes necessary for testicular steroidogenesis, which promotes the survival of germ cells [54]. Any disruption of testicular steroidogenesis could lead to reduced testosterone levels and sperm abnormalities, which are among the factors contributing to male infertility [55]. The World Health Organisation (WHO) classified infertility as a disease of the reproductive system. It is a medical condition characterised by the inability to conceive a pregnancy despite engaging in frequent unprotected sexual intercourse for 12 months or more [47].

Testicular steroidogenesis is primarily mediated by Leydig cells, which are responsible for synthesising testosterone and other androgens in response to luteinising hormone (LH). Testosterone plays a crucial role in the development and maintenance of male reproductive function. This steroid hormone is essential for developing secondary sexual characteristics, regulating libido and modulating spermatogenesis [56]. Spermatogenesis is a well-known process in producing the male gonad, which produces sperm. Furthermore, the production of mature sperm is also closely dependent on androgens like testosterone within the testis. Therefore, in the absence of testosterone or its receptor, spermatogenesis cannot progress beyond the meiosis stage, leading to male infertility [57].

Despite its well-established significance in male reproductive health, the relationship between testosterone levels and male fertility remains complex and multifaceted. Research has demonstrated that optimal testosterone levels are critical for the maintenance of sperm production and quality [57], which is also essential for ensuring male fertility and contributing to a healthy pregnancy. However, hypoandrogenism (low testosterone levels) and hyperandrogenism (excess testosterone) conditions can negatively impact fertility. This shows that it is important to maintain normal testosterone levels to ensure male fertility. Furthermore, external factors, such as age, environmental exposures, lifestyle choices and underlying medical conditions, can influence testosterone levels and, consequently, reproductive health [58,59,60].

### 5.1. Testicular Steroidogenesis Dependent on Hypothalamic–Pituitary Regulation

The testis is a complex endocrine organ primarily responsible for controlling steroidogenesis. The testis is inhabited by Leydig cells, which produce the androgen hormone known as testosterone. This hormone is also necessary for the foetus’s growth and maturation during foetal development. During the masculinisation programming window, the foetal testes begin to produce testosterone, which facilitates male gonadal differentiation and development. Spermatogenesis and the maintenance of secondary sexual function depend on testosterone [61]. According to Jayasena et al., the amount of testosterone in the intratesticular cavity is over 100 times more than in the systemic circulation [62]. Testicular steroidogenesis, which involves a series of steroid hormones, triggers a cascade of signalling pathways that mediate the synthesis of testosterone [63,64].

In testicular steroidogenesis, cholesterol is converted into biologically active steroid hormones through a multienzyme process. Several factors affect steroidogenesis, including trafficking and substrate availability, as well as the levels of steroidogenic enzymes. The control of steroidogenesis depends critically on the hypothalamic–pituitary–gonadal (HPG) axis. Gonadotropin-releasing hormone (GnRH) secreted by the brain causes the pituitary gland to release LH. When LH binds to the G protein-coupled LH receptor (Lhcgr) in the Leydig cell membrane, it activates adenylate cyclase and increases the synthesis of intracellular cyclic adenosine 3′,5′-monophosphate (cAMP). Following binding, downstream protein kinase A (PKA) is activated by cAMP. The steroidogenic acute regulatory (StAR) protein is phosphorylated by PKA, resulting in the formation of pStAR. This active version of the StAR protein is necessary for carrying the cholesterol from the outer mitochondrial membrane (OMM) into the inner mitochondrial membrane (IMM) [11,63,65].

A fundamental lipid molecule, cholesterol, is either transferred to the cells by circulating lipoproteins like high-density lipoprotein (HDL) or synthesised in the cells from acetate. The endoplasmic reticulum can synthesise cholesterol, but the primary source of this precursor for steroidogenesis is the uptake of cholesteryl esters from HDL by scavenger receptor class B type 1 (SR-B1) [63]. SR-B1 is crucial for maintaining the equilibrium of cholesterol levels. Vesicular and non-vesicular routes are used to transport cholesterol to the mitochondria. By acting on the outer mitochondrial membrane, cholesterol-binding proteins like pStAR allow for the non-vesicular transport of cholesterol by inducing the transfer of cholesterol via the transduceosome, a multiprotein scaffold located within the mitochondria. Translocator protein (TSPO), a cholesterol-binding protein present in the OMM, is one of the mitochondrial and cytosolic proteins that make up the transduceosome [11]. Through the aqueous intermembrane gap, this multiprotein complex effectively transports cholesterol from the outer mitochondrial membrane to cytochrome P450scc (CYP11A1) located on the matrix side of the IMM. Endosomes and lysosomes, through vesicular membrane fusion, increase the transport of cholesterol directed towards the mitochondria [66]. CYP11A1 in the mitochondria then converts the cholesterol to pregnenolone.

Specialised membrane contact sites called mitochondria-associated membranes (MAMs) are involved in transporting pregnenolone from the mitochondria to the endoplasmic reticulum [67]. Lipids and tiny compounds can be transferred directly between the OMM and the endoplasmic reticulum membrane at these specialised locations [67]. Pregnenolone is converted to testosterone via the Δ4 or Δ5 routes in the endoplasmic reticulum by the enzymes hydroxysteroid dehydrogenases (HSDs) and cytochrome P450 (CYP), whereas the Δ5 pathway is used in humans to convert pregnenolone to testosterone. The 17α-hydroxylase activity of CYP17A1 hydroxylates pregnenolone to 17α-hydroxypregnenolone [63]. Subsequently, CYP17A1 uses its 17,20-lyase activity to transform 17α-hydroxy pregnenolone into dehydroepiandrosterone (DHEA). DHEA is then converted to androstenediol and androstenedione by 17β-hydroxysteroid dehydrogenases (17β-HSDs) and 3β-hydroxysteroid dehydrogenase (3β-HSD), respectively. Both enzymes are also involved in the conversion of androstenediol and androstenedione into testosterone. Testosterone can be converted into its more powerful form, known as dihydrotestosterone (DHT), via 5α-reductase [64,68].

### 5.2. Testicular Steroidogenesis (Independent HPG Axis) and the Involvement of GPRC6A

Recent studies have revealed the potential involvement of ucOCN in testicular steroidogenesis, a critical process in male reproductive health. ucOCN specifically acts on Leydig cells, as demonstrated in several experimental studies [9,54,69,70]. Nevertheless, studies have yet to report other mesenchymal cell types with the same ability as osteoblasts to increase testosterone formation [69]. Furthermore, it has been shown that ucOCN does not enhance steroid sex hormone production in the ovaries of rats. Aside from this, the ucOCN also does not increase the aromatase activity in Leydig cells, which in turn are unable to induce oestrogen production. Moreover, ucOCN has been demonstrated to influence systemic energy metabolism, which may indirectly affect steroidogenesis. By regulating insulin sensitivity and glucose metabolism, ucOCN could create a favourable environment for Leydig cell function. Evidence suggests that ucOCN levels correlate with testosterone levels in men, indicating a potential regulatory feedback loop where ucOCN supports testosterone production while testosterone influences the release of ucOCN from osteoblasts [71].

Previous in vivo and human studies have found that ucOCN binding to GPRC6A influences testosterone production [72], while a reduction in ucOCN can affect male fertility [1,9,69]. An in vivo study reported that OCN knockout mice have smaller litter sizes, reduced testis weight, oligospermia and low testosterone levels [50,73]. In addition, male mice in the exon II GPRC6A KO strain were found to be more feminine, as evidenced by their smaller testicles, lighter seminal vesicles and decreased genitoanal distance. However, a study performed by Moriishi and Komori [74] found no significant changes in the testis weights, serum testosterone levels, number of spermatozoa, frequencies of abnormal spermatozoa, germ cell apoptosis, testis and epididymis morphology, or gene expression, which are necessary for testosterone biosynthesis for Ocn–/– mice compared to wild-type mice [74]. Hence, further study is necessary to determine the physiological role of the receptor, which is regrettably still unknown due to a general lack of agreement or validation of the reported phenotypes of the various KO mice [75].

ucOCN has been discovered to have a positive correlation with blood testosterone concentration using patient-based samples from bone illnesses and population-based data; individuals with lower ucOCN levels also had lower testosterone levels and vice versa. In a previous study, 190 subjects (74 oligozoospermic, 58 azoospermic patients and 58 normozoospermic subjects acting as controls) referred to an infertility centre in Syria had greater serum concentrations of ucOCN, which was found to be related to increased testosterone levels in the serum. The researcher proposed that the ucOCN-GPRC6A axis, which is a subsidiary of the HPG axis, is involved in the regulation of testosterone production by the Leydig cells in the testis [71]. In addition, Matta et al. found elevated levels of ucOCN in men with primary late-onset hypogonadism (LOH) and subclinical hypogonadism (SCH) [69]. Additionally, ucOCN was positively correlated with LH, LH/T, oestradiol and SHBG and negatively correlated with the free androgen index (FAI) in all subjects. Elevated ucOCN levels in patients with late-onset hypogonadism may represent a compensatory mechanism for impaired Leydig cell function and increased aromatisation, attempting to adjust testosterone levels.

The binding of ucOCN operates through a bone–testicular axis independent of the hypothalamic–pituitary–testicular (HPT) axis [69] or functions as an enhancer of testosterone synthesis in response to gonadotropin stimulation [72]. GPRC6A is found to be the principal receptor involved in the testicular steroidogenesis-independent HPT axis. Accordingly, a recent study by Jawich et al. [71] found a strong indication that the ucOCN/GPRC6A axis is involved in the regulation of testosterone production. It has been demonstrated that ucOCN, via its putative receptor GPRC6A, stimulates the synthesis of testosterone by Leydig cells in mice concurrently with the LH-mediated HPG axis [71].

GPRC6A is a cation-sensing G protein-coupled receptor involved in stimulating testosterone secretion in Leydig cells. Based on research employing three distinct global GPRC6A knockout mice models, where exon II, exon VI or the entire locus had been deleted, several physiological roles of GPRC6A were proposed, such as glucose metabolism, energy homeostasis, gastrointestinal nutrient-sensing receptor, bone homeostasis and male fertility [75]. The ucOCN receptor GPRC6A was strongly expressed in the Leydig cells of the testis [72]. It was demonstrated that the binding of ucOCN can independently regulate steroidogenesis without involving the HPG axis. The GPRC6A receptor is linked to adenylate cyclase, which leads to an increase in cAMP production. This activation triggers signalling pathways and ultimately activates downstream effectors like CREB. CREB then stimulates the expression of various genes that encode important steroidogenic proteins and enzymes, such as StAR, CYP11A1, CYP17A1 and 3HSD [70]. Overall, ucOCN represents a novel factor in the complex regulation of testicular steroidogenesis. Nonetheless, further research is needed to fully elucidate the mechanisms by which ucOCN influences Leydig cell function and to explore its potential as a therapeutic target for conditions related to male reproductive health.

**Figure 2:**
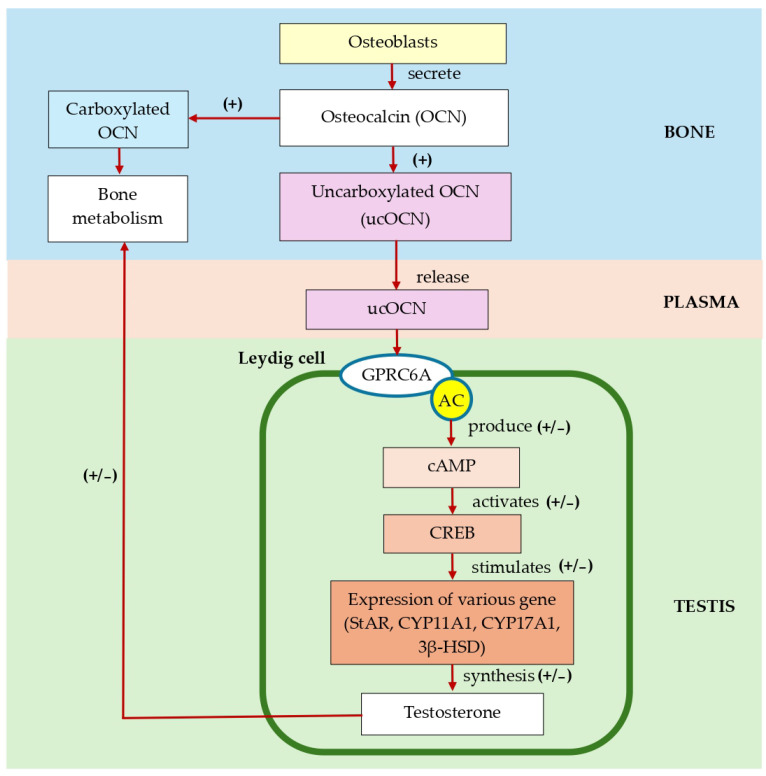
The proposed action of osteocalcin (OCN) and its receptor, G protein-coupled receptor (GPRC6A), between bone and the testis. In bone, osteoblasts secrete OCN, the majority of which becomes fully carboxylated. In addition, decarboxylation of OCN (ucOCN) occurs during bone resorption. Due to its action in reducing the affinity for the bone matrix, ucOCN is released into the plasma. Plasma ucOCN may bind to surface receptor GPRC6A on the membrane of Leydig cells, thus activating adenylate cyclase (AC), which generates cAMP from ATP. Increasing cAMP may activate signalling pathways and downstream effectors, including CREB, thus upregulating steroidogenic genes such as StAR, CYP11A1, CYP17A1 and 3β-HSD. The expression of these genes enhances the production of testosterone, which positively affects bone metabolism.

The potential role of OCN in testosterone synthesis is illustrated in Figure 2. The action of OCN and its receptor, GPRC6A, links bone and testis functions. Osteoblasts in bones secrete osteocalcin (OCN), which undergoes post-translational modifications to form its carboxylated and undercarboxylated forms. The binding of ucOCN to the GPRC6A receptor in Leydig cells in the testes leads to the upregulation of steroidogenic genes like StAR, CYP11A1, CYP17A1 and 3β-HSD. These genes promote testosterone production, which in turn influences bone metabolism positively. Future research should focus on elucidating the molecular mechanisms of ucOCN and GPRC6A signalling in bone and the testis to explore their impact on bone metabolism, testosterone production and overall health, which could be beneficial for future therapeutic strategies.

## 6. Conclusions

In conclusion, the action of OCN and its receptor GPRC6A highlighted the potential role of OCN in male reproductive and bone health. OCN not only stimulates testosterone production in Leydig cells and contributes to male reproductive function but also plays a significant role in metabolic regulation, thus influencing insulin sensitivity and energy metabolism. This review has explored the possible dual function of OCN not only in promoting bone health but also in influencing testicular function and male fertility. Furthermore, it has been postulated that OCN may have anti-ageing properties, potentially mitigating age-related declines in testosterone levels and improving metabolic health. This finding has implications for conditions such as prostate cancer, where the role of testosterone is complex and nuanced, necessitating further exploration into how OCN might influence tumour biology. Future research should focus on the underlying mechanisms governing the effects of OCN, as well as its potential therapeutic implications in treating conditions related to bone and reproductive health. By elucidating the complex relationship between bone metabolism and testicular function, researchers can pave the way for innovative strategies to enhance male reproductive health and address associated disorders.

## Data Availability

Not applicable.
